# A randomised clinical trial of high-intensity focused ultrasound ablation for the treatment of patients with localised breast cancer

**DOI:** 10.1038/sj.bjc.6601411

**Published:** 2003-12-09

**Authors:** F Wu, Z-B Wang, Y-De Cao, W-Z Chen, J Bai, J-Z Zou, H Zhu

**Affiliations:** 1 Institute of Ultrasonic Engineering in Medicine, and Clinical Center for Tumor Therapy of 2nd Hospital, Chongqing University of Medical Sciences, Box 153, 1 Medical College Road, Chongqing 400016, China

**Keywords:** high-intensity focused ultrasound, focused ultrasound surgery, breast carcinomas, therapy, ablation

## Abstract

High-intensity focused ultrasound (HIFU) is a noninvasive treatment that induces complete coagulative necrosis of a tumour at depth through the intact skin. This study was to explore the possibility of using HIFU for the treatment of patients with localised breast cancer in a controlled clinical trial. A total of 48 women with biopsy-proven breast cancer (T_1–2_, N_0–2_, M_0_) were randomised to the control group in which modified radical mastectomy was performed, and the HIFU group in which an extracorporeal HIFU ablation of breast cancer was followed by modified radical mastectomy. Short-term follow-up, pathologic and immunohistochemical stains were performed to assess the therapeutic effects on tumour and complications of HIFU. The results showed that no severe side effect was observed in the HIFU-treated patients. Pathologic findings revealed that HIFU-treated tumour cells underwent complete coagulative necrosis, and tumour vascular vessels were severely damaged. Immunohistochemical staining showed that no expression of PCNA, MMP-9, and CD44v6 was detected within the treated tumour cells in the HIFU group, indicating that the treated tumour cells lost the abilities of proliferation, invasion, and metastasis. It is concluded that, as a noninvasive therapy, HIFU could be effective, safe, and feasible in the extracorporeal treatment of localised breast cancer.

Breast cancer is the most common malignancy in women and, each year, more than 1 million new cases of breast cancer are diagnosed worldwide (McPherson *et al*, 2000). Radical and modified radical mastectomy including axillary lymph node dissection has long been regarded as appropriate therapies. During the last two decades, significant advances have been made in the development of early detection modalities and therapeutic methods. Breast conservation surgery, combined with radiotherapy, chemotherapy, and hormonal therapy, is performed with increasing frequency in patients with early-stage breast cancer. The more from the mastectomy toward breast conservation therapy has not changed long-term survival rates of patients with breast cancer (Veronesi *et al*, 1993; Fisher *et al*, 1995; Jacobson *et al*, 1995; Curran *et al*, 1998).

In spite of this progress, much remains to be achieved, and major new therapies will be required to keep on the fight against breast cancer. Recently, cryoablation (Staren *et al*, 1997), laser (Robinson *et al*, 1998; Dowlatshahi *et al*, 2000), radiofrequency (Bohm *et al*, 2000; McGahan *et al*, 2000), as minimally invasive modalities, have been studied to explore the possibility of ablating breast lesions. However, these alternative treatments require at least percutaneous access and only destroy small lesion in the management of breast tumours.

High-intensity focused ultrasound (HIFU) is a noninvasive technique for the thermal ablation of solid tumours. Ultrasound (US) beam can be focused and transmitted through solid tissues within the body. This allows the possibility of using an extracorporeal source of US for therapeutic purpose. With real-time image guidance, HIFU could noninvasively induce complete coagulative necrosis of a target tumour, without requiring surgical exposure or insertion of instruments into the lesion. These advantages make it one of the most attractive potential therapies for the localised treatment of tumour. To our knowledge, most of the studies on HIFU have been dealing with animal experiments in which HIFU has induced target lesions at depth in tumour tissues and normal organs, including liver, kidney, prostate, bladder, and soft tissue (Chapelon *et al*, 1992; Chen *et al*, 1993; Sibille *et al*, 1993; Prat *et al*, 1995; Rowland *et al*, 1997).

Up to now, in the clinical application of HIFU ablation for human breast tumour, Hynynen *et al* (2001) reported that 11 breast fibroadenomas in nine patients were treated with HIFU. Eight of the 11 lesions treated with HIFU demonstrated complete or partial ablation response. No adverse effects were detected, except for one case of transient oedema in the pectoralis muscle 2 days after therapy. Huber *et al* (2001) used MRI-guided HIFU to treat a 56-year-old patient with breast cancer. Postprocedural pathologic examination indicated that HIFU induced lethal and sublethal tumour ablation without damage to the surrounding healthy tissue or systemic effects. However, to our knowledge, no randomised clinical trial exists that explores the efficacy and safety of HIFU in the treatment of patients with breast cancer, with emphasis on the assessment of histological changes in the treated tumour. In this paper, we studied the efficacy and safty of HIFU treatment in a randomised controlled trial for the ablation of patients with localised breast cancer. The purpose of this study was to investigate the efficacy, safety, and feasibility of HIFU ablation in the treatment of patients with localised breast cancer. This paper reports the work in progress on this noninvasive modality, and it is not our intention to compare it with other breast conservation therapies for the treatment of patients with early breast cancer at present.

## MATERIALS AND METHODS

### HIFU therapeutic system

**Figure 1:**
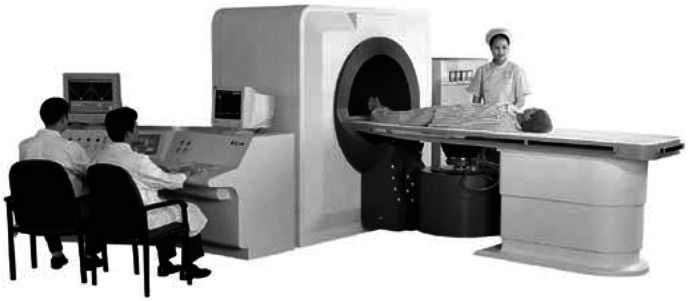
JC-HIFU therapeutic system for tumour (Chongqing HAIFU™ Technology Company, People's Republic of China).

As described in detail previously (Wu *et al*, 2001a, 2002), the HIFU therapeutic system used in this clinical trial of breast cancer treatment principally consists of a diagnostic US device, units for computer automatic control, six-direction movement, and a therapeutic planning system, a US generator, integrated US therapy transducers, and a degassed water circulation unit (see Figure 1

). The therapeutic US beam is produced by a 12-cm diameter PZT-4 piezo-ceramic transducer with a focal length of 90 mm, operating at a frequency of 1.6 MHz. The focal region is ellipsoid, with dimensions of 3.3 mm along the beam axis and 1.1 mm in the transverse direction.

An AU3 US imaging device (Esaote, Genoa, Italy) was used as the real-time imaging unit of the system. This imaging probe (3.5–5.0 MHz) is situated at the centre of the HIFU transducer for real-time guidance during HIFU procedure. The US beams of the therapeutic transducer and the imaging probe completely overlap, so that the longitudinal axis of HIFU beams is in the 2D US imaging plane. The integrated transducer is attached to electric motors, and can be moved smoothly in the six directions with millimetric precision. Through computer control, the imaging transducer was placed either against the skin or at a distance from the skin in the water for pretreatment imaging. However, during HIFU ablation, the imaging probe was usually situated in the water apart from the skin.

### Patients

A total of 48 women with biopsy-proven breast cancer were selected for this clinical trial phase II. Before the beginning of this study, the protocol design was approved by the ethics committee at our university. In accordance with the specification stipulated by the Helsinki Committee, an informed consent form was signed by each patient at the time of enrollment after the principles of HIFU thermal ablation had been completely described. The selection criteria were as follows: histologically proven invasive breast cancer (T_1-2_, N_0-2_, M_0_); single palpable tumours no greater than 6 cm in diameter; the lesion boundaries visualised with color Doppler US imaging, circumscribed at least more than 0.5 cm from skin or rib cage, and more than 2 cm from nipple. All patients were older than 18 years and had no breast implants. They had stable haematogenic parameters, and no history of active myocardial infarction within the past 6 months. They were randomised to two treatment groups: the control group (*n*=25), in which modified radical mastectomy was performed without any intervention prior to surgery; or the HIFU group (*n*=23), in which extracorporeal *in situ* ablation of the breast cancer with HIFU was followed by modified radical mastectomy within 1–2 weeks. A 2-week follow-up after HIFU treatment was performed to evaluate the potential side effects of HIFU such as skin burns, local pain or discomfort, mammary oedema, haemorrhage or infection, and fever. After surgery, all patients received relevant doses of local radiation therapy, conventional chemotherapy, and hormonal therapy, as part of their adjuvant treatment.

Preoperative clinical assessments included the patient’s history, a physical examination, haemotology tests, electrolytes, renal, and liver function tests. Color Doppler US imaging of the diseased breast, with a 10 MHz linear array probe (Q-2000, Siemens, Erlangen, Germany), was performed by a radiologist. Direct visualisation of blood flow within the lesion was imaged by adjusting color setting for optimal slow flow detection. If a patient was recruited to receive the thermal ablation on the basis of the images, US-guided core needle biopsy was performed to confirm histological diagnosis. Technetium bone scanning and a chest radiograph were required for the each case. A comparison of the eligible patients’ characteristics in both groups, including age distribution, tumour size, histologic diagnosis, node status, and TNM classification, is shown in Table 1

**Table 1:** Characteristics of eligible patients in both groups

**Characteristics**	**Control group**	**HIFU group**
No. of patients	25	23
Age (years)	45.5±1.2	46.5±1.7
Tumour diameter (cm)	3.5±0.23 (1.8–5.6)	3.1±0.79 (2.0–4.7)
		
*Histology*		
Invasive	21	21
Noninvasive	4	2
		
*Node status*		
N_0_	13	12
N_1_	6	5
N_2_	6	6
		
*TNM*		
I	2	2
II	22	21
III	1	0

. Three of 23 patients in HIFU group received one pre- and postprocedural magnetic resonance imaging (1.0-T scanner, Impact, Siemens, Erlangen, Germany). The MRI protocol included transverse conventional spin echo (SE) T1-weighted image (WI), FSE T2-WI with fat saturation and dynamic contrast-enhanced (Gadolinium, 0.2 ml kg^−1^, Magnevist, Berlex Laboratories, Wayne, USA). Two radiologists reviewed the pre- and postprocedural MR imaging, and reached a consensus in each patient.

### HIFU 3D conformal treatment

In this study, real-time US imaging was utilised to monitor the HIFU ablation procedure. It can accomplish three separate functions, including real-time targeting of the tumour to be treated, guidance of US energy deposition within the treated region, and rapid real-time assessment of the volume of coagulation necrosis during therapy. High-intensity focused ultrasound treatment was performed in the patients under intravenous sedation (*N*=4) or general anaesthesia (*N*=19). During and following suitable anaesthesia, the patient was monitored to track the blood pressure, pulse, respiration rate, temperature, and peripheral oxygenation. Then, the patient was placed prone and carefully positioned, so that the skin overlaying to the lesion to be treated was easily in contact with degassed water.

The coaxial US imaging device was used to establish the 3D image of the whole tumour. For therapeutic purposes, the whole tumour was divided into slices with 5 mm separation using US images. By scanning the HIFU beam in successive sweeps from the deep to the shallow regions of the tumour, the targeted regions on each slice were completely ablated. This process was repeated slice by slice to achieve complete tumour ablation, in a manner resembling that of cutting away slices of bread. In this study, the extent of HIFU treatment was larger than the tumour extent, in order to ensure the ablation of any adjacent microsatellites and to obtain a sufficient tumour-free margin. It included the breast lesion and its marginal breast tissue about 1.5–2.0 cm around the visible tumour. This is a principle that is routinely employed in conventional surgery. During the HIFU treatment, each slice of US images taken before and immediately after the treatment of each slice were compared. These differences between treated and untreated areas were used for monitoring the therapeutic effect. In this study, the target tissue was exposed at acoustic focal peak intensities from 5000 to 15 000 W cm^−2^. The scanning speed ranged 1–3 mm s^−1^, and the track length was 20 mm. High-intensity focused ultrasound treatment time ranged from 45 min to 2.5 h (median, 1.3 h).

### Pathologic examination

In the clinical trial, modified radical mastectomy was performed in all of the eligible patients. In all, 23 patients in HIFU group underwent the operation within 1–2 weeks after the thermal ablation. All removed specimens including breast tumour and normal breast tissue were evaluated by gross and histological observations. They were serially sectioned at approximately 5 mm intervals and the slice thickness was measured. Gross observations were recorded in a protocol containing the overall appearance, size, and shape. By using a calipers, the visible lesions on each slice were measured in both parallel and perpendicular axes, and volume was calculated by multiplying the area by slice thickness. Representative sections were prepared for routine microscopic analysis. They were fixed in 10% phosphate-buffered formalin (pH=7), embedded in paraffin, cut at distances of 1 mm in 4 *μ*m-thick slices, and stained with haematoxylin and eosin (H&E). To study the possibility of tumour vascular wall destruction induced by HIFU ablation, elasticity fibrin and collagen fibrin double staining (Victoria blue and ponceau's histochemical staining) were performed.

### Immunohistochemistry

Immunohistochemistry was performed in all removed breast tissue from eligible patients. Proliferating cell nuclear antigen (PCNA), cell adhesion molecule CD44v6, and matrix metalloproteinase-9 (MMP-9) are molecular indicators that represent malignant behaviours of breast cancer cell such as proliferation, invasion, and metastasis, respectively. They were identified using the biotin–streptavidin–peroxidase immunohistochemical technology in treated breast cancer in the HIFU group, breast cancer in the control group, and normal breast tissue from the HIFU group, respectively. The paraffin-embedded breast specimens were sectioned in 5-*μ*m-thick slices.

The slices were deparaffinised in xylene, and rehydrated. Endogenous peroxidase was blocked with 0.3% hydrogen peroxide in methanol for 20 min. Antigen retrieval was performed using microwave heating. Subsequently, sections were preincubated with 1% bovine serum albumin. The primary antibodies used were mouse monoclonal anti-human PCNA, anti-CD44v6, and anti-MMP-9. All of them were obtained from Santa Cruz Biotechnology (Santa Cruz, CA, USA). Sections were incubated at room temperature with three kinds of the primary antibody, respectively, followed by biotinylated second antibody incubation. The chromogen was 3,3-diaminobenzidine tetrahydrochloride (brown). Sections were incubated with phosphate-buffered saline, which served as a negative control instead of the primary antibody.

The number of immunostained cells was counted in a microscopic grid, 0.5 × 0.5 mm^2^ in size (0.25 mm^2^), using a microscopic field of × 200. A total of 10 areas with the most abundant distribution were chosen in each case for this observation. Two independent observers judged the distribution of these three variables and counted the positive cells. If the results were inconsistent, the two observers discussed to reach a consensus on the semi-quantitative scoring. For the PCNA, we used the labelling index of PCNA in cancer cells to evaluate the result. The number of PCNA^+^ cancer cells was counted among 100 cancer cells using a × 200 microscopic field.

### Statistical analysis

All observed data are displayed as the mean value plus or minus the standard deviation. The statistical significance of any observed difference between the mean values of control and treatment groups was evaluated using an unpaired Student's *t*-test. The variability in data was compared with the F test for variance. The differences in percentage data were analysed by using the Fisher's exact test. Statistical significance was defined as a *P*-value of less than 0.05.

## RESULTS

### Short-term follow-up

**Figure 2:**
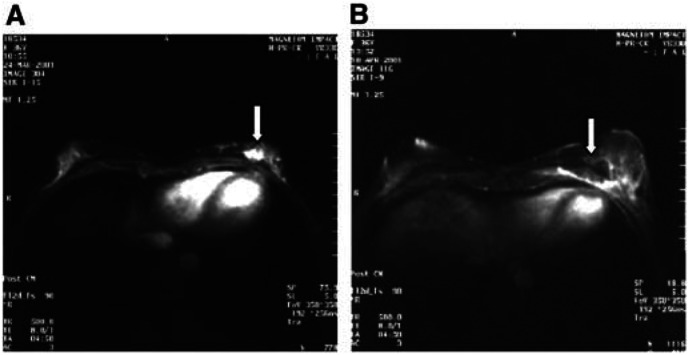
Contrast-enhanced MRI of a left breast cancer (arrowed) in a 36-year-old woman before (**A**) and 1 week after (**B**) HIFU ablation. Note the absence of contrast uptake in ablated volume, including tumour and a margin of treated normal breast tissue about 1.5–2.0 cm around the cancer. Oedema is observed in the mammary tissue surrounding the treated region.

Among 23 patients, 16 cases underwent surgery at 7–10 days after HIFU ablation, and the remaining seven patients were operated at 11–14 days. After HIFU, oedema was noted in the mammary tissue surrounding the treated tumour. In all cases, the treated area included the tumour and a 1.5–2.0 cm margin of normal breast tissue around the tumour. At 7–10 days postoperatively, the oedema gradually disappeared. In total, 14 patients experienced mild local pain, warmth, and sensation of heaviness in the diseased breast. But only four patients were given 3–5 days prescription for oral analgesics. One patient had minimal skin burn, which had entirely recovered by 10 days post-HIFU. There was no incidence of bleeding or infection of the treated breast requiring intervention. Three patients received one MRI examination after HIFU treatment. Compared with preprocedural images, enhanced MRI revealed the absence of contrast enhancement in the treated region including tumour and 1.5–2.0 cm normal breast tissue surrounding the tumour (see Figure 2

), indicative of coagulative necrosis.

### Pathologic examination

**Figure 3:**
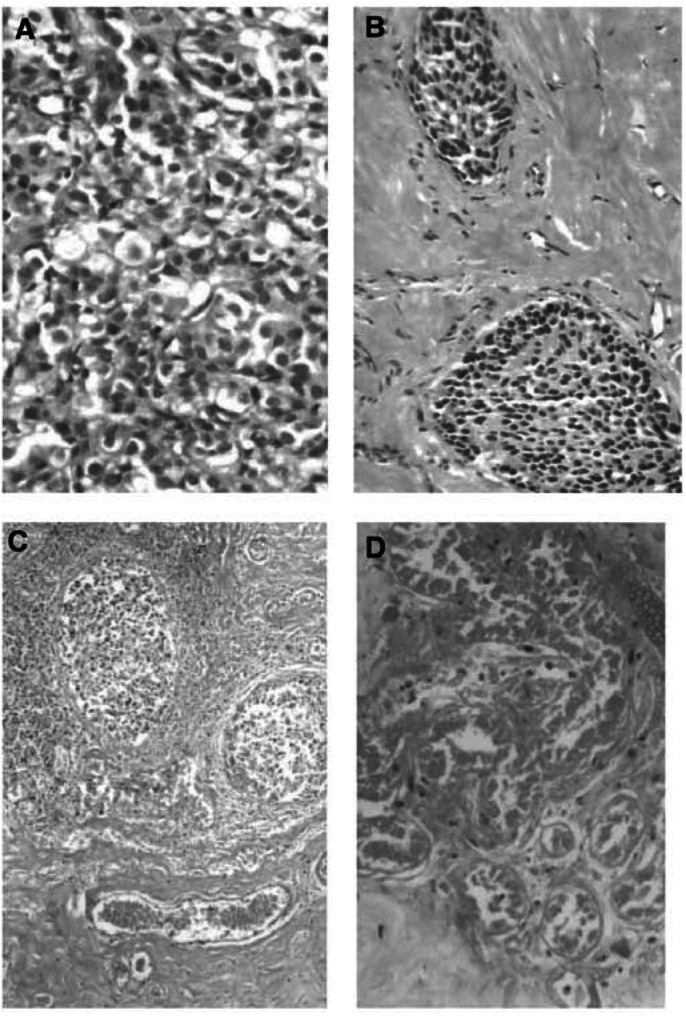
Microscopic changes of tumour cells in patients with breast cancer treated by HIFU. (**A**) Untreated viable breast cancer cells. (**B–D**): Necrosed tumour cells represented by pyknotic nuclei (**B**); nuclear disruption (**C**); and nuclear disappearance (**D**). H&E staining × 400.

**Figure 4:**
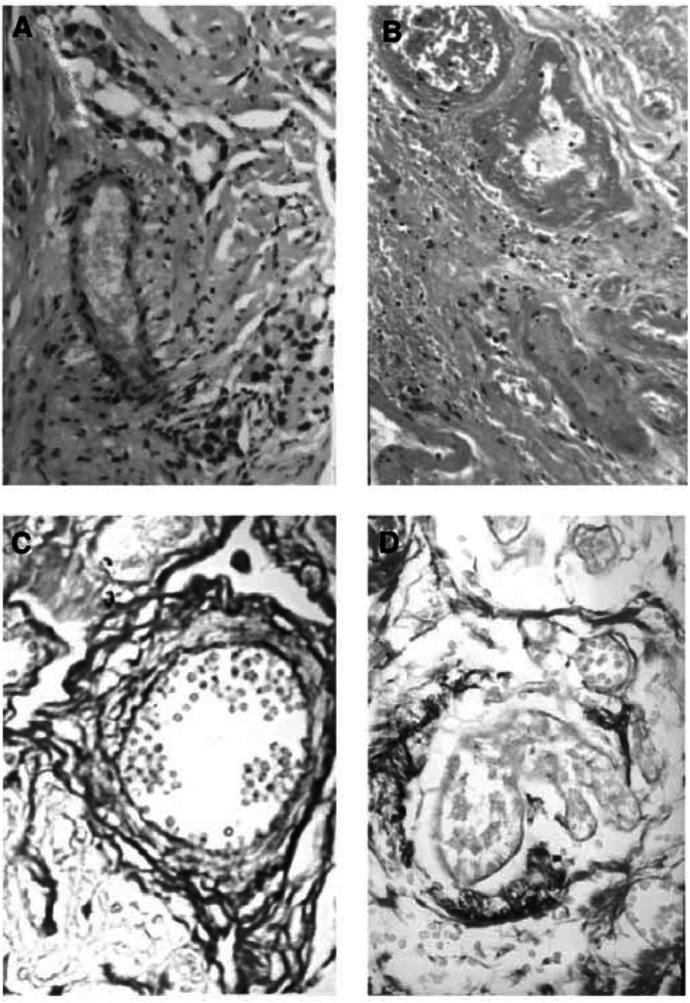
Microscopic changes of tumour vascularity in patients with breast cancer treated by HIFU. (**A**) Untreated tumour blood vessels; (**B**) destruction of tumour vascularity with disruption of the endothelium and tunica media, H&E staining × 400. (**C**) untreated tumour vessel wall; (**D**) destroyed tumour blood vessels with the collapse and disruption of vascular elasticity fibrin and collagen fibrin, Victoria blue and ponceau's histochemical staining × 400.

All treated tumours in the 23 patients were confirmed by gross observation after removal of the diseased breast. Neither bleeding of the treated regions nor injury of intervening tissues was identified, indicating the safety of HIFU ablation. Macroscopic examination showed that HIFU treatment induced complete coagulative necrosis of the target tissue, which included the tumour and a mean margin of 1.80±0.58 cm (range from 1.5 to 2.2 cm) of an obviously normal breast tissue surrounding the tumour. This felt firmer on palpation than normal tissue. At the margin between the treated and untreated regions, there was a rim of congestion that represented an inflammatory reaction to thermal ablation. Histological examination revealed homogeneous coagulative necrosis, including the tumour and normal breast tissue within the target region. Figure 3

shows that the tumour cells of breast cancer treated with HIFU were very abnormal, with the appearance of irreversible cell death, as demonstrated by pyknotic nuclei, nuclear disruption, and disappearance. Variable amounts of granulation tissue were noted with the presence of immature fibroblasts, inflammatory cells, and new capillaries in the boundary region. Compared with blood vessels in the control group, vascular structure in the HIFU group was seriously damaged. Appearances were of indistinct cellular margins, endothelial disruption, and nuclear disappearance, and destruction of tunica media, indicative of coagulative necrosis (Figure 4

). The size of the destroyed vessels was less than 2 mm in diameter, including the arteries and veins. Scattered intravascular thrombi were often observed in the destroyed vessels. Victoria blue and ponceau's histochemical staining showed that vascular elasticity fibrin and collagen fibrin were significantly collapsed and disrupted, as shown in Figure 4.

### Immunohistochemical staining

Figure 5

**Figure 5:**
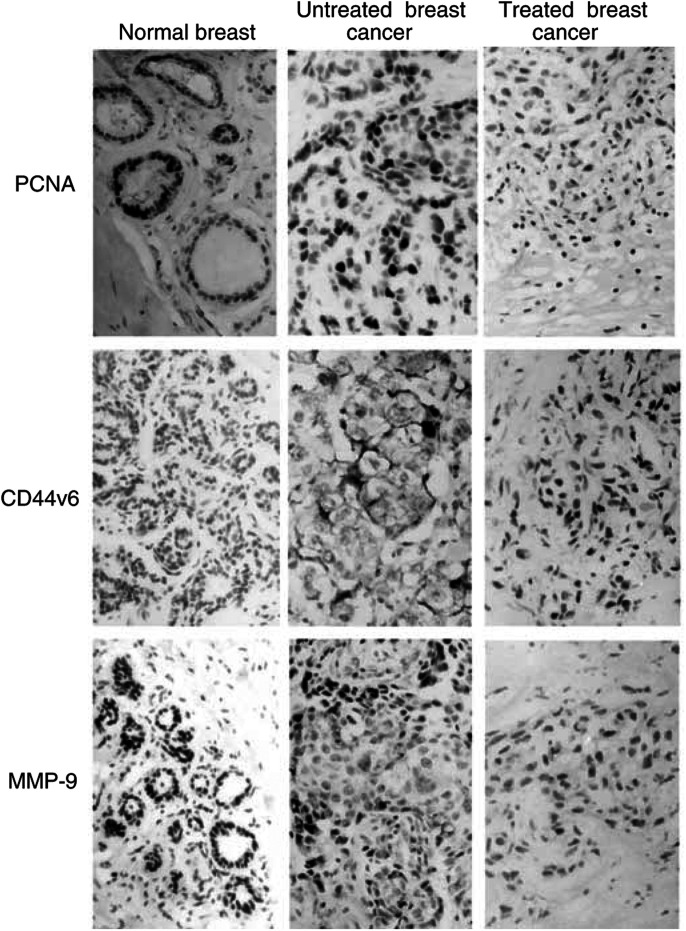
Immunohistochemistry for PCNA (the first row), CD44v6 (the second row), and MMP-9 (the third row) in the patients with breast cancer treated by HIFU (S-P staining, × 400). The immunoreactivity is shown by the color brown. From left to right, each separated morphology is represented by a normal breast tissue in the HIFU group, untreated cancer cells in the control group, and treated cancer in the HIFU group, respectively. Compared with untreated cancer cells, no positive cells of PCNA, CD44v6, and MMP-9 within the treated cancer cells were observed in the HIFU group.

showed immunohistochemical staining for PCNA, CD44v6, and MMP-9 in breast cancer in the control group, normal mammary tissue, and treated breast cancer in the HIFU group, respectively. The percentages of the PCNA labelling index in the normal breast tissue, breast cancer, and HIFU-treated tumour were 6, 44, and 0%. CD44v6-positive cells were present in 56% of tumour samples in the control group (*n*=14), and 4.5% of normal breast tissue (*n*=1). But, in the HIFU group, CD44v6-positive cells were not detected within the treated breast cancer (*n*=0). CD44v6^+^ cell density was significantly higher in tumour than in the normal breast tissue (*P*< 0.01), but in the HIFU group the proportion of these membrane-positive cancer cells was 0%. Compared with 60% of MMP-9 within breast cancer in the control group (*n*=15), no MMP-9^+^ cancer cells were detected in either normal breast cancer or treated breast cancer (*n*=0), as shown in Table 2

**Table 2:** Results of immunohistochemical staining in both groups

	**PCNA labelling index (%)**	**CD44v6 (%)**	**MMP-9 (%)**
Normal breast tissue	6	4.5	0
Untreated breast cancer	44	56	60
Treated breast cancer	0	0	0

.

## DISCUSSION

The energy deposition of focused US on the target tissue causes coagulative necrosis of tumours (Chapelon *et al*, 1992; Chen *et al*, 1993; Sibille *et al*, 1993; Prat *et al*, 1995; Rowland *et al*, 1997). In this study, pathological examination revealed that both breast cancer cells and normal tissues within the treated region presented a typical appearance of coagulative necrosis with severe nuclear damage. Furthermore, tumour vessels were severely damaged in the patients with breast cancer, resulting in significant vascular disruption and vessel occlusion. By using immunohistochemical staining, we found that no expression of PCNA, CD44v6, and MMP-9 of the treated tumour cells was identified in the HIFU group, unlike those in the control group. These findings indicated that the treated breast cancer cells presented had not only undergone coagulative necrosis in histology, but also the absence of malignant behaviours such as proliferation, invasion, and metastasis.

The goal of our clinical study is to explore the efficacy, safety, and feasibility of extracorporeal HIFU ablation for primary breast cancer. To achieve this purpose, we hypothesise that both tumour and some normal breast tissue surrounding the tumour should be completely destroyed with focused US energy. This larger ablation extent would guarantee an adequate ‘surgical’ HIFU margin surrounding the tumour, and would be required if HIFU therapy is considered as an alternative to surgical procedure. This principle is routinely used in conventional surgery in order to ensure the removal of adjacent microsatellites, and to allow for uncertainty that frequently exists concerning the exact location of definite tumour margins. In the clinical study, histological examination revealed that HIFU induced coagulative necrosis of the target tumour and its surrounding normal tissue by about 1.5–2.2 cm (mean 1.80±0.58 cm), which corresponded closely with the pretreatment extent (1.5–2.0 cm around the tumour) determined in the therapeutic protocol before HIFU procedure. This result indicated that, with real-time US guidance, HIFU can precisely cause a complete necrosis of target tissue, including the tumour and normal tissue.

In clinical applications, real-time imaging modalities including both US (Vallancien *et al*, 1996; Beerlage *et al*, 1999; Chapelon *et al*, 1999; Gelet *et al*, 1999; Wu *et al*, 2001a, 2002; Uchida *et al*, 2002) and MRI (Huber *et al*, 2001; Hynynen *et al*, 2001) can guide HIFU ablation of solid tumours. These imaging techniques have been used for targeting the lesion to be treated, monitoring the therapeutic procedure, assessing the thermal effects on target tissue, and controlling the ultrasonic energy. In this study, the reasons for choosing real-time US for the guidance of HIFU treatment were as follows: low cost, flexibility, speed of image processing, and extensive availability. During the HIFU procedure, obvious changes in grey scale within the target tissue were immediately detected on the US image after HIFU ablation. Our previous studies indicated that these hyperechoic zones corresponded well to the extent of coagulative necrosis, and that there was a close relationship between the extent of necrosis as measured by gross examination and the hyperechoic extent measured on the US image immediately after FUS in both *in vivo* and *in vitro* tissues (Wang *et al*, 1997; Wu *et al*, 1998, 2000, 2001b; Bai *et al*, 1999). In the clinical treatment of patients with breast cancer, we also found that the hyperechogenic regions were regular in shape and size, and conformed to the extent of coagulative necrosis induced by HIFU ablation (Liu *et al*, 1998; Cao *et al*, 2001; Wu *et al*, 2001; Zhou *et al*, 2001). Although the mechanism for tissue grey-scale change is still unclear, US effects on tissue such as heating and cavitation are involved in the formation of coagulative necrosis, and real-time diagnostic US guidance is seen to be useful in the detection of the coagulative necrosis of target tissue during HIFU procedures.

As a noninvasive method, HIFU has a potential application in the treatment of patients with breast cancer. However, some problems still exist with this technique. First, to our knowledge, neither MRI nor diagnostic US have satisfactory sensitivity for precise visualisation of tumour margins. A completely precise imaging modality for pretreatment demonstration of tumour extent is not currently available. Therefore, in our clinical study, image-detected breast cancer and 1.5–2.0 cm normal tissue surrounding the cancer were completely ablated to allow for the uncertainty that frequently exists concerning the exact location of definite tumour margins. However, for some superficial lesions close to the skin, there is a possibility of causing skin burns, or leaving residual viable tumour cells if the overlying skin is left undamaged. A second problem is that a palpable firm lump would persist in the treated region for a period of time after HIFU treatment. This lump may not be satisfactory or cosmetically desirable for the patient, although the tissue would revert to benign status. This could also cause potential problems with follow-up images, in distinguishing whether there is local recurrence postoperatively. Finally, a suitable indication of HIFU ablation for breast cancer is essential if this technique can be used in clinical practice. Breast tumours with undefined margins, scattered multiple foci, and tumours close to the nipple should be excluded as potential targets for HIFU.

In conclusion, this study demonstrates for the first time that US-guided HIFU is effective, safe, and feasible in the treatment of localised breast cancer with well-demarcated margins. However, much supplementary investigation is necessary to explore these above problems, and large controlled clinical trials must be performed before extracorporeal HIFU can be recommended as a conserving breast modality in the treatment of breast cancer.
